# Selection of Construction Methods: A Knowledge-Based Approach

**DOI:** 10.1155/2013/938503

**Published:** 2013-12-24

**Authors:** Ximena Ferrada, Alfredo Serpell, Miroslaw Skibniewski

**Affiliations:** ^1^Department of Construction Engineering and Management, Catholic University of Chile, Vicuña Mackenna 4860, Macul, 7820436 Santiago, Chile; ^2^Center of Excellence in Project Management, Department of Civil & Environmental Engineering, A. J. Clark School of Engineering, 1188 G. L. Martin Hall, University of Maryland, College Park, MD 20742-3021, USA

## Abstract

The appropriate selection of construction methods to be used during the execution of a construction project is a major determinant of high productivity, but sometimes this selection process is performed without the care and the systematic approach that it deserves, bringing negative consequences. This paper proposes a knowledge management approach that will enable the intelligent use of corporate experience and information and help to improve the selection of construction methods for a project. Then a knowledge-based system to support this decision-making process is proposed and described. To define and design the system, semistructured interviews were conducted within three construction companies with the purpose of studying the way that the method' selection process is carried out in practice and the knowledge associated with it. A prototype of a Construction Methods Knowledge System (CMKS) was developed and then validated with construction industry professionals. As a conclusion, the CMKS was perceived as a valuable tool for construction methods' selection, by helping companies to generate a corporate memory on this issue, reducing the reliance on individual knowledge and also the subjectivity of the decision-making process. The described benefits as provided by the system favor a better performance of construction projects.

## 1. Introduction

Given the impact construction methods have on productivity, quality, and cost, their selection is a key decision for the proper development of a construction project, and it is one of the main factors affecting the productivity and efficiency of construction projects [1]. Also, it is considered as one of the five potential areas of productivity loss according to the European Construction Institute (ECI) [2]. These facts highlight the significance of an appropriate selection of construction methods for a project since deficient methods for executing the work can cause significant losses of productivity on site [3].

Then, how can this selection of construction methods be done correctly? This paper proposes to address this decision problem from the perspective of knowledge management (KM). The implementation of this approach is particularly appealing for the construction sector [4] and is a powerful tool that can help this industry to innovate and improve its performance [5, 6].

Construction companies have difficulties in the management of the information and knowledge associated with construction projects, combined with the fact that much of information about previous projects is not reused because there are not adequate mechanisms for its storage [7]. In addition, the knowledge created in the field is not usually shared, which tends to produce its loss [8]. This situation eventually affects decision-making processes because correct decisions are the result of the careful management and analysis of the information and knowledge available [9].

This paper presents a knowledge management approach that includes both a KM application framework and a prototype system developed to verify the framework of the KM approach. The objective of the system is to support decision-making for the correct selection of construction methods for a construction project. The next sections present the main background on the selection of construction methods and knowledge management, the conceptual development and main features of the proposed knowledge system, and, finally, the operation of the prototype system used to validate the proposal.

## 2. General Background

### 2.1. Selection of Construction Methods

Construction methods are the means used to transform resources into constructed products [10]. According to Illingworth [11], programming and management techniques are of little value for a project if construction methods are not the most optimal in terms of cost or are not safe to run. The selection of construction methods affects not only the selection of the activities and their work sequence, but also its duration [12]. In construction, this process is highly iterative and requires the construction team to examine a variety of data sources as well as tap into its own experience base to formulate a set of efficient methods [13]. In cases like this, when a decision problem has at least two conflicting criteria and at least two solution criteria, the problem is considered a multicriteria decision analysis [14].

### 2.2. Knowledge Management in the Construction Industry

It was in the mid-80s that people began to appreciate the increasingly prominent role of knowledge in the competitive environment [15], with the emergence of knowledge-based organizations [16]. This new approach recognizes the knowledge as one of the most valuable assets of an organization away from the traditional economic view, which recognizes knowledge as something external to the company and with no connection to the economic process [17]. Moreover, this approach gives a clear structure to manage knowledge, with greater emphasis on the knowledge itself and with a hierarchy above information and data [18]. Thus, it is possible to define knowledge management as the way in which organizations create, capture, and use knowledge to achieve organizational objectives [9].

Construction companies obtain most of their knowledge from the projects they undertake. However, the knowledge generated within each project is finally stored in reports that remarkably few read or is lost because the people involved move to a new project, leave the company, or retire [19, 20], taking with them not only their tacit knowledge, but also a potential source of competitive advantage.

Regarding how to manage knowledge in the selection of construction methods, Ferrada and Serpell [18] indicate that construction companies use the knowledge of individuals to carry out this process. There is not an organizational-based learning process that allows acquiring the relevant knowledge.

Knowledge management in the construction industry is a focus of different types of research work, for example, studies that have tried to understand how to implement knowledge management in construction companies and also the perceptions of people about this topic [4, 5, 21, 22]. Others have focused on developing ontologies and classification systems [23–26].

Learning has also undergone some studies [27–29] as well as the development of knowledge management models [30, 31], the development of systems to store and share knowledge [32], and the development of knowledge maps [33–37].

Other lines of research have focused on understanding the impact of technology in data capture in the field [38], in the management of documentation [39, 40], and in the development of methodologies for the capture and reuse of the knowledge created in projects [19, 41]. Other researchers have studied how to share tacit knowledge within communities of practice [42] and how to make a live capture and reuse of project knowledge [43]. The importance of collaborative knowledge management has also been addressed [44], and in recent years there have been studies about the use of mobile technologies in construction [45], among others.

## 3. A Knowledge Management Approach for the Selection of Construction Methods in Construction Projects 

To study how the selection of construction methods is currently carried out in the local industry, a methodology based on case studies was selected because if there is a relationship between the phenomenon under study and its context, this technique is considered an appropriate research strategy [46, 47]. Then, three Chilean construction companies participated in the research.  Table 1 details companies and professionals interviewed in each case. Data was collected using semistructured interviews. The organizational model of the CommonKADS methodology for developing knowledge management systems [48] was used as a reference for preparing the interviews.

Results of the case studies show that the selection of construction methods is largely based on the previous experience of professionals. It is a process characterized by the complexity of the analysis, the high dependence on individual experience and teamwork, and the need for expert knowledge. Companies' senior management recognizes the need for a structured system to allow a better management of their knowledge by storing it correctly and making its employment less difficult. In addition, knowledge acquisition is not part of an appropriate process, so people have no obligations or incentives to participate in this activity. This situation was highlighted as one of the main barriers for organizational learning about construction methods.

An important part of this research focused on the identification of knowledge gaps in the process of selecting construction methods. The case studies reveal that main gaps exist in the activities “*search for construction methods*” and “*application of the decision criteria.*” Regarding the first one, interviewees indicated that people have an extremely limited time for this activity. Furthermore, individual's knowledge is a fundamental input of this activity considering that there is not a database of stored lessons learned, nor are there procedures for their effective management. Related to the application of the decision/selection criteria, a critical activity for the adequate project performance, it currently depends heavily on the decision maker intuition, and then, decisions are not comparable across projects. Thus, it becomes necessary to reduce the subjectivity and variability of the decision-making process by making it explicit about the most influential decision criteria for selecting construction methods. Results from interviews allowed identifying the key criteria to use in the selection of construction methods, which include project duration, cost, product characteristics, construction method characteristics, and environmental characteristics. The criterion “product characteristics” has two associated subcriteria: build volume and quality requirements, while the criterion “characteristics of the construction method” has five associated subcriteria: familiarity with the construction method, health and safety, level of automation of the method, level of interference with other operations, and availability of the method. Finally, the criterion “environmental characteristics” has four subcriteria associated: location and access, climate, obstacles/topography, and available space. These criteria were validated with experts of the studied companies and used for the development of the knowledge system for the selection of construction methods.

Based on the results of the case studies, the proposed approach for the knowledge system incorporates both knowledge management techniques and technologies. Knowledge management techniques are applicable since there is already a valuation of collaboration and team work in construction companies. The consistent application of these techniques should encourage the creation and transmission of the knowledge associated with the selection of construction methods. For this process, different techniques might be used as follows:

- brainstorming, a technique for generating ideas and creating knowledge that helps to solve problems;
- formal instances of knowledge acquisition, which can be of two types, (1) project-related such as team meetings or final project meetings and (2) associated with the selection of construction methods itself, such as meetings with experts, suppliers, or meetings to discuss problems during the execution of a methodology and determine any possible solution;
- interaction face to face, valued in organizations and that should be encouraged in a structured way;
- research and development transfer meetings, since construction methods relate directly to innovation.

Regarding knowledge management technologies, the information and knowledge gained were stored in organizational databases associated with a knowledge portal called Construction Methods Knowledge System (SCMC in its Spanish acronym). This knowledge portal on a web platform provides easy access from any location and has the capacity of storing in databases all information associated with construction methods. Furthermore, a decision-making support system for the selection of construction methods was accessible from this portal.

The information was stored in the form of construction methods sheets. Each sheet (Table 2) contains the knowledge linked to the selection of construction methods as identified in the case studies. Thus, each sheet focuses not only on the technical aspects of each method, but also on two issues that are of importance in the development of the process: (1) the selection of subcontracts and (2) the search for experts, whether internal or external. All this facilitates the study of each construction method as this information will be stored in one place—the organizational database—saving time and effort in the search.

Different aspects were considered to design the final construction method sheet. First, apply the same construction method to different projects. Second, store information in the construction methods sheet using a unified format. Third, the sheet should be simple and easy to fill. Fourth, allow an overview of the construction method, and include a list of experts who may be contacted in case more detail is required. Fifth, indicate if lessons learned about the method exist and if so, show them and allow their download. Sixth, indicate the degree of automation [10], risk level and degree of interference with other operations, and features that might be measured using a scale 1–5, where 1 indicates the lowest automation value for the analyzed item and 5 represents the highest value.

For the decision-making support system, the knowledge related to decision criteria was acquired in meetings with experts on construction methods selection, realized during the case studies as previously indicated.

### 3.1. Prototype Design and Construction

#### 3.1.1. System Requirements

For the design and development of a system, it is necessary to know its requirements, which can be of two types: functional and nonfunctional. Functional requirements are inputs, outputs, processes, and data stored needed to satisfy the system improvement objective, while nonfunctional issues are a description of other features, characteristics, and constraints that define a satisfactory system [49]. Main functional requirements of the system have to do with its ability to store and deploy construction methods sheets, allow finding these sheets within the database, and edit and delete them as necessary. They also highlight the need for the system to upload and download files and to be accessible via the Internet. Main nonfunctional requirements include the need for different types of users, the possibility to upload files in Word or pdf format, the option to export construction methods sheets to MS Excel, and the need to view the system properly using common browsers.

Based on the requirements defined for the system, the computer applications that compose the SCMC were selected. This study began with the search of computer programs available in the market for each of the two principal components of the SCMC: (1) the knowledge portal and (2) the system to support decision making. This task was carried out to determine if appropriate software was available in the market in order to reduce the programming work or to start all programming from scratch if needed.

Regarding the knowledge portal, there was a wide variety of software available in the market, including Alfresco, TikiWiki, and MS Office SharePoint, to name just a few. The evaluation of these software packages considered the analysis of various factors, such as system applications number and type, the allowance of modification of their programming code, and their cost. Finally, the best option was to design and construct the system from scratch so that needs of construction companies would be met. For the decision support system, developing software was the least suitable alternative, because commercial software such as Expert Choice and Make it Rational offered what was exactly needed for this part of the prototype. The online system Make it Rational was selected for this purpose, because it is easy to use and allows access through the web. This software uses the Analytic Hierarchy Process (AHP), one of the most widely applied multiattribute decision making methods [50]. The basic idea of this method is to convert subjective assessments of relative importance to a set of overall scores or weights [50]. AHP uses quantitative comparisons to select the preferred alternative by comparing alternatives in pairs, based on their relative performance with respect to a criterion.

#### 3.1.2. System Modeling and Its Architecture

**Figure 1:**
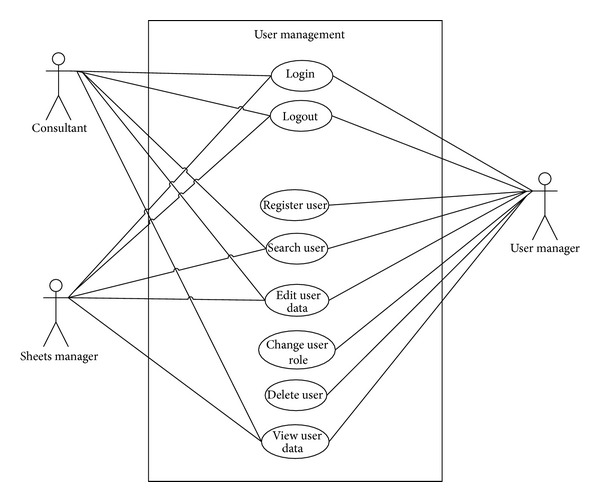
User management in SCMC use-case diagram.

The first step for modeling the system corresponded to developing use cases.  Figure 1 shows an example of the use-case diagram developed specifically for the SCMC user management. The system involves three types of users: User Manager, Sheets Manager, and Consultant. Users link to graphical representations of cases of use, indicating what their different roles within the system are. To develop the system, thirteen use cases were built. Each one was developed in greater detail in order to define the requirements of the prototype clearly.

**Figure 2:**
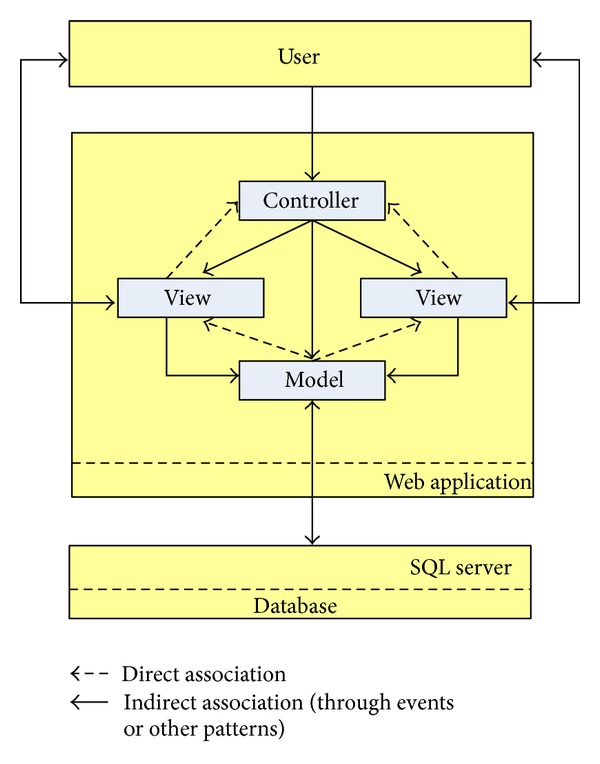
System architecture.

The database system works in Microsoft SQL Server, which is a database management system based on the relational model. When defining the system architecture, the software architecture pattern Model-View-Controller (MVC) was used as shown in Figure 2. This pattern allows separating the data from an application, the user interface, and the business logic into three distinct components [51]. For example, for the prototype system, the domain model recognizes two main entities that shape the SCMC, sheets and users, which also distinguishes two types of interaction on the entities: user management and sheets management. In this case, the sheets are the description of a construction method used in a given context, while users correspond to the representation of individuals who access the system and can manage records according to their role. The views, meanwhile, are in charge of showing the user the information contained in the model, presenting it in a form suitable for interaction [51]. Usually this is the user interface. The controller is responsible for directing the control flow of the application due to external messages, such as data entered by the user or menu items selected by him [52]. From these messages, the controller is responsible for the modification of the model or opening and closing views [52].

The design of the system's graphic interfaces was made with a free HTML template selected for this purpose (colors, organization of content, fonts, etc.) and located inside the MVC application.

#### 3.1.3. Construction Methods Knowledge System (SCMC)

**Figure 3:**
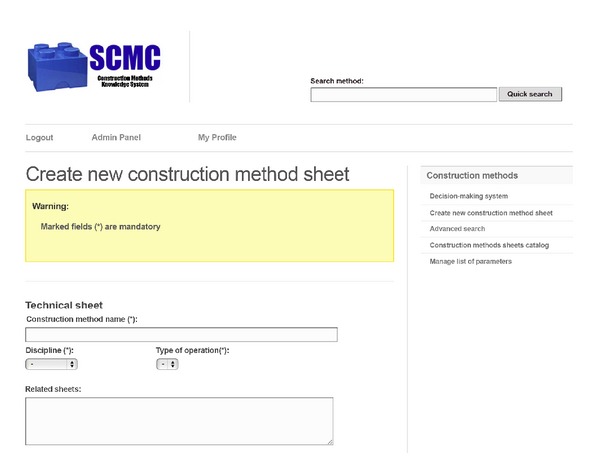
Sheet creation for a new method.

**Figure 4:**
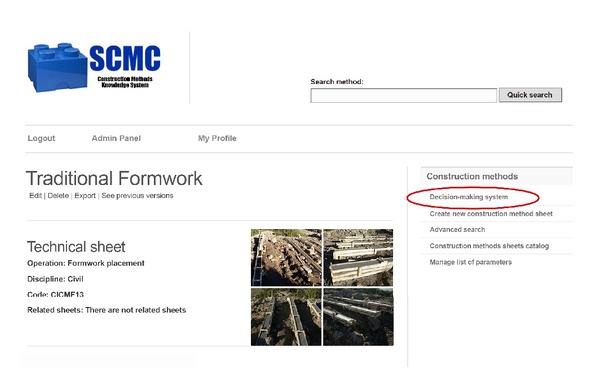
Completed method sheet.

Access to the SCMC is through the Internet, with a login and password. After the authentication, the user accesses the system according to his role: User Manager, Sheets Manager, or Consultant. To illustrate how the system works, look at the case of a Project Manager that has a Sheets Manager role. If this user wants to enter information about a new construction method, he or she will face a view as presented in Figure 3. There, he/she must enter at least the mandatory data to create a new sheet: method's name, discipline, operation type, risk level, yield, cost, core activities, and whether the method has been used previously in the company. Once the Sheets Manager saves the new file, the system shows the new sheet with options to edit, delete, export, or view previous versions of the sheet (see Figure 4).

**Figure 5:**
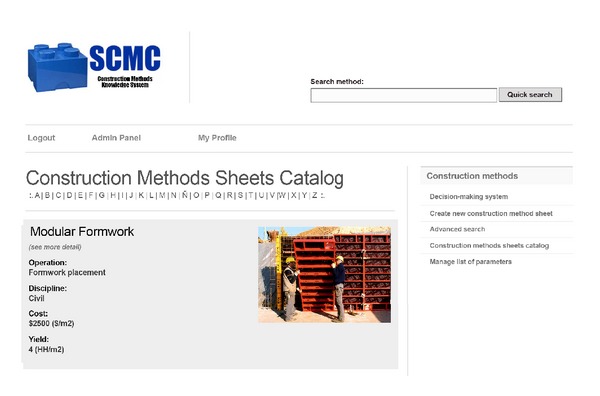
Catalog of construction methods and sheets search.

The search for construction methods sheets can be performed in three ways (Figure 5): (1) by using a quick search feature, which allows searching by keywords; (2) looking into a catalog of methods, which allows searching by the initial letter of the name of the method; or (3) through an advanced search, which allows searching using filters such as the method name, discipline to which it belongs, and operation type. In this case, the system searches all the sheets on the database by means of the fields defined by the user and present the results that match the search parameters.

Results appear as in Figure 5. Once the user receives the results provided by the system, he or she can access the full version of the sheets that he or she wants to review in more detail. After this revision, he or she can define the feasible alternatives to perform the operation under consideration. With this information, the user can request quotes, conduct a cost-benefit analysis, and select the two or three most feasible options considered for the project. To carry out the final selection of the construction method it is necessary to evaluate these alternatives in terms of different decision criteria. In order to make this part of the process more objective, the SCMC includes among its core components a system to support decision-making. The link for accessing this system is on the right side of the screen (Figure 4).

**Figure 6:**
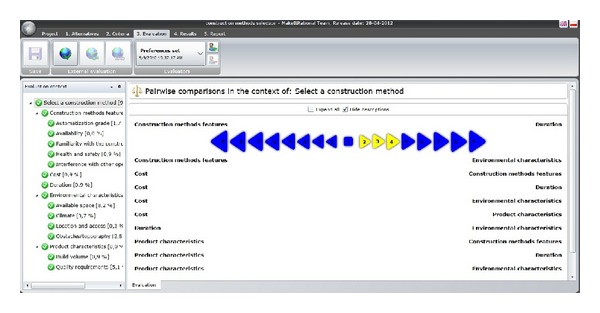
Input of user's preferences using Make it Rational.

When accessing the application, a file named “*Selection of construction methods*” should open, which contains the hierarchical structure of decision criteria obtained from the case studies. When the file opens, the user faces a set of windows: (a) ALTERNATIVES, (b) CRITERIA, (c) EVALUATION, (d) RESULTS, and (e) REPORT. The first window allows defining the alternatives for the decision process. With this information the user enters the CRITERIA window, which contains the decision criteria previously defined and the description of each one. The third window allows the input of the user's preferences as described in the AHP methodology. For this, three kinds of comparisons are necessary. First, for each subcriterion or criteria without subdivision, different alternatives are compared by pairs (Figure 6) and preferences of the user are requested. The user enters his/her preference by marking the triangle containing the number that better represents it, ranging from 1 to 9. Also, for each criterion with division, the user must assess the importance of each subcriterion with respect to the central criterion, also in pairs. Finally, main criteria should be compared among them with respect to the ultimate goal, which is the selection of a construction method.

**Figure 7:**
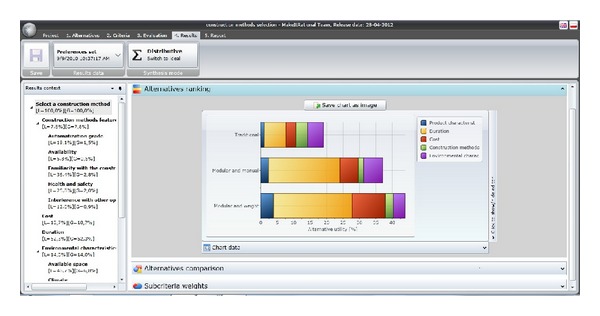
View of results in Make it Rational.

Once all comparisons are made, it is possible to access the “RESULTS” window. The system indicates what alternative is the best in terms of the user's preferences. For example, Figure 7 shows a bar graph with the ranking of alternatives. This graph shows the utility of each alternative for the decision maker. Finally, if the user wishes, in the REPORT window, a report with the results can be automatically generated and then exported to RTF, PDF, Excel, HTML, and XPS format.

The decision making support system allows the decision maker to select the most optimal construction method to perform the operation studied objectively, having analyzed all the criteria that could affect this decision, which decreases subjectivity and organizes perceptions and judgments. This analysis can also increase the likelihood of success in implementing the construction method in the field and force a detailed analysis of all factors that affect the decision, which directly impacts the performance of the project.

## 4. Validation of the Prototype System

During the development of the system, the SCMC was presented three times to two experts on construction methods selection, each from a different construction company. The comments received were used to modify some aesthetic aspects of the system and improve its interaction with the user. A final validation of the construction methods selection system was carried out with a wider group of experts. The goal of this activity was to verify the usefulness of the system and its practical applicability, even if it has not been used yet in the field.

The validation process included interviews with eleven construction professionals from six different companies, with experience in construction methods' selection. Two of these professionals had participated in progress meetings of the SCMC; five had participated in the case studies without involvement in the development of the SCMC and four were integrated at this final stage. These professionals work in the following roles: Technical Manager, Head of the Estimation Department, Project Manager, and Head of Management and Innovation. All of them received a complete presentation of the SCMC. After it, each professional was interviewed briefly in order to know his/her opinions about the prototype.

Interviewees considered the system as a useful tool for the selection of construction methods because it helps to make more informed decisions and provides all the information needed in just one place. Furthermore, with the same level of importance, people said that the system is a valuable mean to increase organizational knowledge, reducing dependence on individual knowledge. Also, the system was considered as a suitable tool for sharing that knowledge within the organization. These results are presented in Table 3.

Closely connected to the benefits from the adoption of the system in construction companies, respondents highlighted the time savings in the search for alternative construction methods and the possibility to store, organize, and classify information regarding these issues. Interviewees also indicated that the system could enhance the competitiveness of the company and that it is a reliable guide for the decision-making process, decreasing the likelihood of making a wrong decision. Likewise, they indicated that this system would help them to develop a knowledge oriented culture in the organization. These results are presented in Table 4.

Ten of the eleven respondents would use the prototype in their daily work and considered it friendly and easy to use. In these cases, what stood out as its main practical value was the increase of their productivity by saving time in searching for alternative methods of construction and the easy access to information. Furthermore, interviewees indicated that this system would help their companies in guiding their decision process for construction methods selection, introducing innovations within the organization and reducing costs and time in projects. Only one interviewee indicated that he would not use the system in his daily work. He explained that projects carried out by his company (mainly high-rise residential buildings) are quite similar between them. Then, in this case there would be no need to select different methods. Also, alternatives methods for the construction of these projects would be very limited. In fact, the interviewee noted that a system with the same characteristics, but much more focused on technical information sheets, would be much more attractive for his company. These results are presented in Table 5.

During the interviews, some stimulating comments emerged in relation to the future implementation of the system. First, there is a concern about how to integrate such a system into the organization. It is believed that young professionals would be more willing to use the system because they are more used to work with computers and software, unlike older professionals. Under this logic, it is logic to think that there would be more resistance to the implementation of the system in more experienced professionals. Furthermore, regarding the difficulties of integrating the system into large companies, it becomes clear that it needs to be part of the policies and long-term objectives of the company, in order to promote its development.

In many cases, there were concerns about the way in which the system would be incorporated in projects and how to ensure that the necessary information is entered. The option of integrating the knowledge management system with the quality system of the company was considered appropriate and useful, given the potential synergy between the two. Other comments mentioned two additional key aspects: the organizational culture and workers' competencies. In order to integrate a real-knowledge management system in an organization, it is vital to develop a culture of knowledge in the enterprise, to recognize the value of sharing experiences, document them and make use of the knowledge of the organization to facilitate everyday tasks. Moreover, even when people intend to participate actively in a knowledge management system, they may not have all the necessary competencies, especially regarding information technologies. In the same way, if workers who will execute a selected method chosen in SCMC do not have the technical skills to carry it out, no matter how meritorious the decision making was, the result will not be as expected. These aspects should be analyzed in more detail by each organization, to determine how to close the gaps that exist today.

## 5. Discussion and Conclusions 

The research found that empirical experience of construction field practitioners is the best source of knowledge for the selection of construction methods. It is highly likely that this situation is repeated in other similar processes. Therefore, people should be careful not to incorporate knowledge systems that merely use information technology for managing knowledge since they only encode explicit knowledge, ignoring the experience-rich tacit knowledge that it is difficult to transfer through information technologies. To avoid this, it is necessary to include appropriate knowledge management techniques. Moreover, when incorporating information technology to a construction company, it should be friendly, intuitive, and simple to use, since otherwise it will not be used.

Also, the most appropriate occasions to acquire knowledge seem to be working meetings, and the mechanisms used to acquire knowledge could be construction methods' sheets and documented lessons learned, as they can capture the knowledge and also part of the context in which it was generated. Construction methods' sheets are a way to standardize the knowledge on construction methods, facilitating in this way the decision-making process. This knowledge is stored in the system database, transforming the individual experience of professionals in organizational knowledge. Records stored in the system will enhance the performance of searching construction methods saving time and effort.

Since the definition of a construction method is a complex process, the best way to organize the knowledge associated with this process is by developing a hierarchy of decision criteria which later serves as the basis for the application of a methodology of decision making with multiple criteria within the knowledge management system. Thus, every option is evaluated based on preestablished criteria, where the decision maker incorporates and evaluates the main requirements of the project, through his or her analysis of preferences.

Opinions given by respondents during the SCMC prototype's validation point out that the prototype could respond appropriately to the needs of construction companies regarding the information and knowledge stored, the contribution to the decision-making process, and its simplicity of use. These features make the system valuable and applicable in the day to day activities of a construction company.

The system could become a tool for supporting the selection of construction methods and improving the quality of these decisions. Beside this, the application of the system will reduce the impact generated by the departure of key employees from the company because their knowledge will be stored and available. In addition, the prototype showed that the proposed knowledge management system offers a concrete way to capture and use the knowledge to improve the selection of construction methods.

## Figures and Tables

**Table 1:** Companies participating in case studies.

Company	Certification	Construction markets	Established in the year	Number of professionals interviewed
A	ISO 9000	Civil, industrial, and building work	1960	5
B	ISO 9000	Residential, institutional, commercial, and tall buildings work	1989	6
C	ISO 9000	Civil, industrial, and building work	1978	3

**Table 2:** Construction methods sheet.

Construction methods sheet			
**Discipline:**		Code:	Related to other sheets:
**Operation:**			
**Construction method:**			
**Degree of automation:**	**Yield: **	**Cost:**	**Risk level:**
**Used in the company: yes/no**		**Degree of interference with other operations: **	
**Restrictions:**		**Restriction type:**	
		Space	Competencies
		Inspections/permits	Weather
		Topographic	Machinery
		Materials	Security

**Type of project where it has been used**		**Project names**	
**Labor requirements**		**Materials requirements**	
**Equipment and machinery requirements **		**Temporary requirements**	
**Fundamental activities**		**Work process**	
**Subcontracts that perform the method**		**Experts on the method**	
**Is there any lesson-learned associated to this construction method?**			

**Table 3:** Utility of the SCMC.

Utility	Frequency
Make decisions with more knowledge	7
Have all the information in just one place	6
Increase organizational knowledge	2
Share knowledge	2

**Table 4:** Major benefits of adoption of SCMC in a construction company.

Benefits	Frequency
Time savings in the search for alternative construction methods	6
Possibility to store, organize, and classify companies' information	5
Increase competitiveness of the company	4
Decrease the likelihood of making a wrong decision	2
Development of a knowledge oriented culture in the organization	1

**Table 5:** Practical values of SCMC.

Practical values	Frequency
Increase productivity	5
Easy access to information	5
Guide decision process	4
Introduce innovations within the organization	2
Reduce cost	1
